# Health-Economic Evaluation of the German Osteoporotic Fracture Prevention Program in Rural Areas (OFRA): Mobility and Falls Prevention Classes, Examination of Bone Health, and Consultation on Safety in the Living Environment

**DOI:** 10.1007/s11606-022-07691-2

**Published:** 2022-07-25

**Authors:** Claudia Konnopka, Gisela Büchele, Dietrich Rothenbacher, Patrick Roigk, Kilian Rapp, Hans-Helmut König

**Affiliations:** 1 Department of Health Economics and Health Services Research, University Medical Center Hamburg-Eppendorf, Martinistr. 52, 20246 Hamburg, Germany; 2 Institute of Epidemiology and Medical Biometry, Ulm University, Ulm, Germany; 3 Department of Clinical Gerontology, Robert-Bosch-Hospital, Stuttgart, Germany

**Keywords:** fragility fractures, frailty, prevention, cost-effectiveness, net-benefit approach

## Abstract

**Background:**

Fragility fractures are one of the leading causes of disability in older adults. Yet, evidence for effectiveness and cost-effectiveness of preventive approaches combining bone health and fall prevention is rare.

**Objective:**

To conduct a health-economic evaluation of the German osteoporotic fracture prevention program in rural areas (OFRA).

**Design:**

Secondary cluster–randomized intervention study based on routine data.

**Participants:**

All districts in five federal states in Germany were cluster-randomized as intervention or control districts. OFRA was offered to community-living (a) women aged 75–79 years or (b) women and men aged 70–84 years with a prior fragility fracture in the intervention districts. Individuals who meet these criteria in the control districts were assigned to the control group.

**Intervention:**

OFRA comprised mobility and falls prevention classes, examination of bone health by bone density measurement, and consultation on safety in the home living environment.

**Main Measures:**

We measured health-care costs and effectiveness in terms of time to fragility fracture or death within 1 year after initial contact, based on health insurance claims data. Implementation costs were recorded by the intervention performers. We calculated an incremental cost-effectiveness ratio (ICER) and employed the net-benefit approach to construct a cost-effectiveness acceptability curve (CEAC).

**Key Results:**

There were 9408 individuals in the intervention group and 27,318 in the control group. Mean time to fragility fracture or death (difference: 0.82 days) and health-care costs (difference: 111.73€, *p* < .01) were reduced, but mean intervention costs (difference: 260.10€) increased total costs (difference: 148.37€, *p* < .001) in the intervention group. The ICER per fracture-free year of survival was 66,094.63€. The CEAC showed no acceptable probability of cost-effectiveness at a reasonable willingness to pay.

**Conclusion:**

OFRA showed reduced rates of fragility fractures, but had high implementation costs, resulting in an unfavorable ICER. The cost-effectiveness of OFRA may improve with a longer follow-up.

## BACKGROUND

Falls in older and fragile adults often result in fragility fractures, which become a major health problem within an aging population.^1^ Fragility fractures not only are associated with considerable costs, but frequently lead to negative health outcomes, such as reduced quality of life, immobility, and mortality.^2^ Due to the ongoing demographic shift in the German population,^3^ the number of fractures attributable to osteoporosis may increase by 238% from 2010 to 2050.^4^ Thus, prevention of fragility fractures is highly relevant.

The two main underlying mechanisms of fragility fracture are osteoporosis and falls.^5^ Regarding falls, there is a large body of evidence on preventive measures for community-living older adults, with the most effective being physical exercise, which may even reduce fractures.^6,7^ To diminish the consequences of falls, improvement of the bone quality by treatment of osteoporosis may reduce the risk of fragility fractures.^8^ Therefore, approaches are recommended to combine bone health and fall prevention.^9^

However, coordinated preventive approaches for community-living older adults that include both fall prevention measures and osteoporosis examination and treatment are limited,^10,11^ and do not exist in Germany at all. Moreover, to the best of our knowledge, there are no studies evaluating a program combining bone health and fall prevention for community-dwelling older adults from a health-economic perspective.

The objective of this study was to conduct a health-economic evaluation of a large fragility fracture prevention program in Germany. The program was carried out in rural areas in Germany from October 2015 through to September 2017, and aimed at improving physical function and reducing the risk of falls and fractures in older adults. The components of the program were exercise classes, examination of bone health by a dual-energy X-ray absorptiometry (DXA) scan, and a consultation on safety adjustments in the living environment. The program was described in detail in the study protocol^12^ and the primary analysis.^13^

## DATA AND METHODS

### Intervention

The osteoporotic fracture prevention program in rural areas (OFRA) was a large health-insurance-driven program to improve safe mobility and reduce the risk of falls and fragility fractures in older adults living in rural areas. The participating health insurance company “Sozialversicherung für Landwirtschaft, Forsten und Gartenbau” (SVLFG) provides health insurance that is compulsory for people working in agriculture, gardening, and forestry, and insures approx. 670,000 persons. The health insurance company cooperated with the German Association of Rural Women (DLV), the German Gymnastics Association (DTB), and the Robert Bosch Institute for Medical Research (RBMF) as part of OFRA.

OFRA consisted of mobility and fall prevention classes, examination of bone health by a DXA bone density measurement, osteoporotic treatment where indicated, and consultation on safety in the living environment.

The mobility and fall prevention classes were based on the Otago exercise program^14,15^ and on a fall prevention program by the DTB.^16^ Six sessions of 90 min were delivered within 6 weeks. Individuals were encouraged to perform exercises at home further on, for which they received an instruction booklet and a training log. In order to increase the spatial proximity for the individuals, classes took place at local facilities nearby the individuals’ home. The trainers were physiotherapists or exercise instructors from local sports clubs provided by the DTB with additional education regarding OFRA. Exercise devices such as weight cuffs were provided by the SVLFG. Participation was open to all individuals, including those not insured at the SVLFG, and free of charge.

A DXA scan for a bone density measurement was recommended to all individuals at no charge based on the German osteoporosis guideline.^17^ The individuals were asked to talk to their general practitioners. The general practitioners were reimbursed for counseling and treatment, if necessary.

Nearly all individuals were visited by a prevention manager of the SVLFG who encouraged them to attend the classes or to make use of a DXA scan. If required, the prevention manager gave advice on how to improve safety of the living environment.

Furthermore, the SVLFG established telecenters which initially contacted and informed selected individuals in the intervention districts about OFRA. Where an individual was interested in participating, the telecenters referred them to one of the classes nearby. The telecenters also coordinated the prevention classes, and sent training materials to the trainers. Further information was made available on a homepage.

The study was registered at the German Clinical Trials Register (DRKS-ID: DRKS00009000) and approved by the ethics committee of Ulm University (proposal 120/15). Individuals gave informed consent for their data to be shared, for example, to the organizer of an exercise class.

### Study Design, Selection Criteria, and Data Sources

The implementation of OFRA took place in 47 administrative districts in five federal states (Baden-Württemberg, Bavaria, Hesse, Lower Saxony, and Rhineland-Palatinate), corresponding to around half of the area of Germany. All administrative districts were randomly assigned to either intervention or control districts using a 1:3 cluster randomization. The control districts received no intervention.

The program was offered to (i) women and men aged 70 to < 85 years with a fragility fracture in the previous 5 years, and (ii) all women aged 75 to < 80 years, who were insured by the SVLFG and community-living in the intervention districts. Individuals were excluded if they were living in a nursing home or if their care need for the basic activities of daily living was 120 min or more according to the categorization of the German long-term-care insurance (until the year 2016: care level 2; since 2017: degree of care dependence 3). All individuals in the control districts with identical selection criteria were categorized as control group (CG).

OFRA started on October 1, 2015 and was offered for 2 years. The time period between the information letter and the start of an exercise class was usually at least 2 months. Therefore, follow-up began 2 months after the first contact of the intervention group (IG) individuals by the SVLFG. All individuals were followed for 1 year, or until fracture or death. For the analysis, each individual from the IG was randomly matched to three individuals from the CG. This matching was stratified by federal state and selection group. For each individual from the CG, a starting date was assigned according to the matched individual from the IG. After the matching, an equal distribution of pre-intervention characteristics was ensured.

Health insurance claims data were provided by the SVLFG. Information on expenditures for OFRA was recorded by RBMF and SVLFG. The mobility and fall prevention classes were continued following the conclusion of OFRA in September 2017. When calculating costs, we assumed the last classes were in December 2017, allowing sufficient time for the IG to participate after initial contact. Costs were also incurred for the telecenter until December 2017.

### Intervention Costs

All costs occurring before the start of OFRA in October 2015 were assumed as preparation costs, and all costs during OFRA (October 2015–September 2017) as operation costs. Both RBMF and SVLFG had expenditures for OFRA, categorized as staff and other costs.

Staff costs were calculated as follows: personnel working time for the coordination of OFRA, cost of the staff in the telecenters, and cost of prevention managers (recorded and multiplied with appropriate salary scales). Additional payments by the employer such as social security contributions were added, if applicable. For the coordination of OFRA by the RBMF, we assumed all employees’ salary scales as E13 (“employees with completed university study or equivalent skills according to their occupation”). For the coordination of OFRA by the SVLFG, we assumed an 80% share of A10 and 20% share of A13 (“higher intermediate civil servant salary scales”) according to the specifications of the SVLFG. For personnel costs for the telecenters as well as for the prevention managers, the civil servant salary scale A9 was assumed. All salary scales were calculated according the Federal Ministry of Finance.^18^

Other costs occurring before the start of OFRA were considered as preparation costs. Material costs for the mobility and fall prevention classes (e.g., exercise devices) were recorded by the SVLFG. Costs for the education of trainers were fixed at 350€ plus usual expenses for traveling. The number of trainers who completed the education was recorded by the SVLFG. Costs for information technology comprised the establishment and maintenance of the homepage and system adjustments for the monitoring of OFRA and were recorded by RBMF and SVLFG. Travel costs for the coordination of OFRA were recorded by the RBMF.

Other costs occurring during OFRA were considered operational costs. Delivery of a mobility and fall prevention class of six sessions was reimbursed with a fixed 700€. The number of classes was recorded by the SVLFG. Costs for the design, print, and distribution of information material such as letters, reports, instruction booklets, and training logs were recorded by the RBMF and the SVLFG. Travel costs during OFRA were recorded by the RBMF.

The mobility and fall prevention classes were open to participation not only for the IG but for all individuals living nearby. Therefore, the share of individuals from the IG actually was lower than 100% and it would be inappropriate to fully take the costs for those classes into account. Therefore, we calculated a share of costs for the IG as follows: Of 9681 IG individuals, 29.6% (*n* = 2866) attended a class.^19^ As recorded by the SVLFG, there were 1733 classes during the period from October 2015 to December 2017 with an average participation rate of 11.2,^20^ which equals *n* = 19,410 class participants. Of those, 2866 belonged to the IG, which is a share of 14.8%. This share was applied to the material, training, and staff costs of the exercise classes. Furthermore, it was applied to the share of personnel working time of the telecenters which was used for tasks related to the classes, e.g., organization or distribution of training materials, which yielded a share of 59.5% for telecenter costs. Costs for the DXA scan were recorded in the claims data (see next section).

### Fracture-Related Health-Care Costs and Effectiveness

In Germany, health insurance is mandatory and provides comprehensive protection against health-care expenses. Around 90% of the population are insured by statutory health insurance, while 10% have opted for private health insurance. Apart from very low and negligible co-payments, health insurance reimburses all expenses of inpatient and outpatient treatment as well as pharmaceuticals and preventive services to health-care providers. As we used claims data provided by the SVLFG, we investigated direct fracture-related health-care costs from the payer perspective for the intervention period and 1-year follow-up. The following cost categories were retrieved from SVLFG claims data: hospital and rehabilitation facilities due to fragility fractures (ICD-10 code S12, S22, S32, S42, S52, S72, S82), DXA scans (calculated as reimbursement defined in OFRA), medication for osteoporosis with bisphosphonates and denosumab (ATC codes: M05BA, M05BB, M05BX), and both inpatient and outpatient long-term care. We could not observe if long-term care was due to a fracture. However, we assume that the distribution of fracture-related and -unrelated long-term care is equal for IG and CG.

All costs were adjusted for inflation using the gross domestic product price index^21,22^ and reported in 2017 Euro.

Effectiveness was measured as time to hospital admission due to fragility fractures (ICD-10 code S12, S22, S32, S42, S52, S72, S82) or death. For both health-care costs and time to fracture or death, individuals were followed up to 1 year from the date of first contact.

### Statistical Analysis

The difference of fragility fractures between groups was tested with logit models. The difference of costs was tested with two-part models with logit models, to test if there were any costs per individual, and subsequent generalized linear models with gamma distribution to estimate the amount of costs. The difference of the time to fracture or death was tested with Cox proportional hazards models.

The incremental cost-effectiveness ratio (ICER) was calculated:

$$ \mathrm{ICER}=\frac{{\mathrm{mean}\ \mathrm{costs}}_{\mathrm{IG}}-{\mathrm{mean}\ \mathrm{costs}}_{\mathrm{CG}}}{{\mathrm{mean}\ \mathrm{effects}}_{\mathrm{IG}}-{\mathrm{mean}\ \mathrm{effects}}_{\mathrm{CG}}}=\frac{\Delta \overline{\mathrm{costs}}}{\Delta \overline{\mathrm{effects}}} $$

The ICER refers to the additional costs generated by OFRA to achieve an additional fracture-free year of survival, compared to usual care. Where OFRA is less costly and more effective, it is cost-effective. However, if OFRA is costlier and more effective, the cost-effectiveness depends on the maximum willingness to pay (WTP).

Cost-effectiveness acceptability curves were constructed to handle the uncertainty of the ICER. This approach reformulates the ICER into a net-monetary benefit (NMB) and considers different maximum WTP. For each WTP, OFRA is cost-effective if the point estimate of the NMB is positive:

$$ \mathrm{NMB}=\mathrm{WTP}\bullet \Delta \overline{\mathrm{effects}}-\Delta \overline{\mathrm{costs}} $$

Since the WTP was unknown, it was iterated from 0€ to 2,500,000€ in steps of 10,000. The iterated NMB was used as dependent variable in a regression model with OFRA as binary independent variable.

The resulting cost-effectiveness acceptability curve (CEAC) describes OFRA’s probability of being cost-effective at different WTPs. OFRA may be considered cost-effective if the probability of being cost-effective is above 95%.

## RESULTS

There were 47 IG districts with 9408 individuals and 139 CG districts with 27,318 individuals (Table 1). The distribution of individuals per federal state and further characteristics between groups were highly similar, which implied good randomization and matching processes. Of the individuals, 89–90% were female, they were on average 78.8 years old, 29–30% had a fragility fracture in the last 5 years, and 88–89% were not care dependent.

**Table 1:** Population Characteristics at or Before Implementation

	Intervention group		Control group	
Number of districts: *n* (%)	47		139	
Number of individuals: *n* (%)	9408	(25.6%)	27,318	(74.4%)
Individuals in federal state: *n* (%)				
Baden-Wuerttemberg	1513	(16.1%)	4534	(16.6%)
Bavaria	4351	(46.2%)	11,974	(43.8%)
Hesse	593	(6.3%)	2171	(7.9%)
Lower Saxony	1971	(21%)	6224	(22.8%)
Rhineland-Palatinate	980	(10.4%)	2415	(8.8%)
Sex: *n* (%)				
Male	994	(10.6%)	2752	(10.1%)
Female	8414	(89.4%)	24,566	(89.9%)
Age: mean (SD)	78.8	(2.5)	78.8	(2.5)
Pre-fracture: *n* (%)	2834	(30.1%)	8017	(29.3%)
Care dependency: *n* (%)				
No	8371	(89.0%)	24,054	(88.1%)
Yes	1037	(11.0%)	3264	(11.9%)

Due to the case-finding approach, not all addressed individuals participated in all OFRA components: 29.6% of addressed individuals participated in exercise classes. Of those, 29.2% took 5 and 54.6% 6 classes.^23^ 16.7% received DXA bone density measurement and 51.8% advice on safety in the living environment.^19^

Intervention costs were 260.10€ per individual. 62.85€ occurred before and 197.25€ during OFRA (Table 2). Cost-driving categories were staff costs for coordination, telecenters, and prevention managers.

**Table 2:** Intervention Implementation Costs

	Full-time equivalents [years]	Costs [EUR]	Costs per individual (*n* = 9681) [EUR]
Preparation costs (before Oct. 2015)		608,517	62.85
Staff costs for coordination	1.87	144,176	14.89
Material costs for mobility and fall prevention classes		17,996	1.85
Education costs for trainers		1989	0.20
Information technology costs for the homepage		72,318	7.47
System adjustments for intervention monitoring^*^		371,369	38.36
Travel costs		669	0.07
Operation costs (Oct. 2015–Dec. 2017)		1,927,811	197.25
Staff costs for coordination	5.76	427,785	42.33
Staff costs for telecenters	13.5	718,352	74.22
Staff costs for prevention manager	9	474,427	49.01
Costs for mobility and fall prevention classes		180,783	18.63
Information material costs		43,339	4.48
Distribution of information material costs		76,123	7.86
Travel costs		7002	0.72
Total costs		2,536,328	260.10

^*^Adjustment to the system of the SVLFG was necessary to monitor the intervention. Adjustments mainly comprised creation of a database to contact and invite individuals to the intervention and to monitor their participation

The proportion of fragility fractures was slightly lower in the IG than in the CG (difference: 0.3%) (Table 3). The highest reduction was for femoral fractures (0.3%, *p* < .01). Fracture-free time of survival was slightly longer in the IG (0.82 days). Mean fracture-related health-care costs were significantly lower in the IG (difference: 111.73€, *p* < .01), driven by hospital and inpatient long-term care costs. Mean costs for DXA scans (difference: 4.88€, *p* < .001) and medication (difference: 5.81€, *p* < .01) significantly increased in IG, as intended by OFRA. When additionally considering intervention costs, mean total costs were significantly higher in the IG (difference: 148.37€, *p* < .001). The ICER per fracture-free year of survival was 66,094.63€.

**Table 3:** Outcomes and Costs After Implementation

	Intervention group		Control group		Difference (SE)		
Fragility fractures: *n* (%)	322	(3.4%)	1021	(3.7%)	− 0.3%		(0.002)
Of the spine	59	(0.6%)	171	(0.6%)	0.0%		(0.001)
Of the pelvis	42	(0.4%)	91	(0.3%)	0.1%		(0.001)
Of the shoulder or upper arm	39	(0.4%)	147	(0.5%)	− 0.1%		(0.001)
Of the forearm	59	(0.6%)	148	(0.5%)	0.1%		(0.001)
Of the femur	91	(1.0%)	362	(1.3%)	− 0.3%	†	(0.001)
Of the lower leg	32	(0.3%)	102	(0.4%)	− 0.1%		(0.001)
Time to fragility fracture or death [days]: mean (SD)	358.56	(36.1)	357.74	(38.6)	0.82		(0.09)
Fracture-related health-care costs per individual [EUR]: mean (SD)	769.07	(3001.4)	880.80	(3568.8)	− 111.73	†	(40.18)
Thereof in hospital	225.32	(1739.9)	261.37	(1745.7)	− 36.05		(22.33)
Thereof in rehabilitation facilities	37.59	(410.1)	41.34	(439.9)	− 3.75		(5.24)
Thereof due to medication	38.18	(179.4)	32.37	(169.3)	5.81	†	(1.96)
Thereof for DXA scans	5.56	(19.5)	0.68	(6.5)	4.88	*	(0.17)
Thereof due to inpatient long-term care	116.01	(1863.0)	181.11	(2513.9)	− 65.09	‡	(31.38)
Thereof due to outpatient long-term care	346.40	(1047.7)	363.92	(1050.9)	− 17.53		(12.68)
Total implementation costs per individual [EUR]: mean	260.10						
Fracture-related health-care and implementation costs per individual [EUR]: mean (SD)	1029.17	(3001.4)	880.80	(3568.8)	148.37	*	(33.08)
ICER [EUR per fracture-free year of survival]	66,094.63						

*SE* standard error, *ICER* incremental cost-effectiveness ratio

*p < 0.001; ^†^*p* < 0.01; ^‡^*p* < 0.05

**Figure 1:**
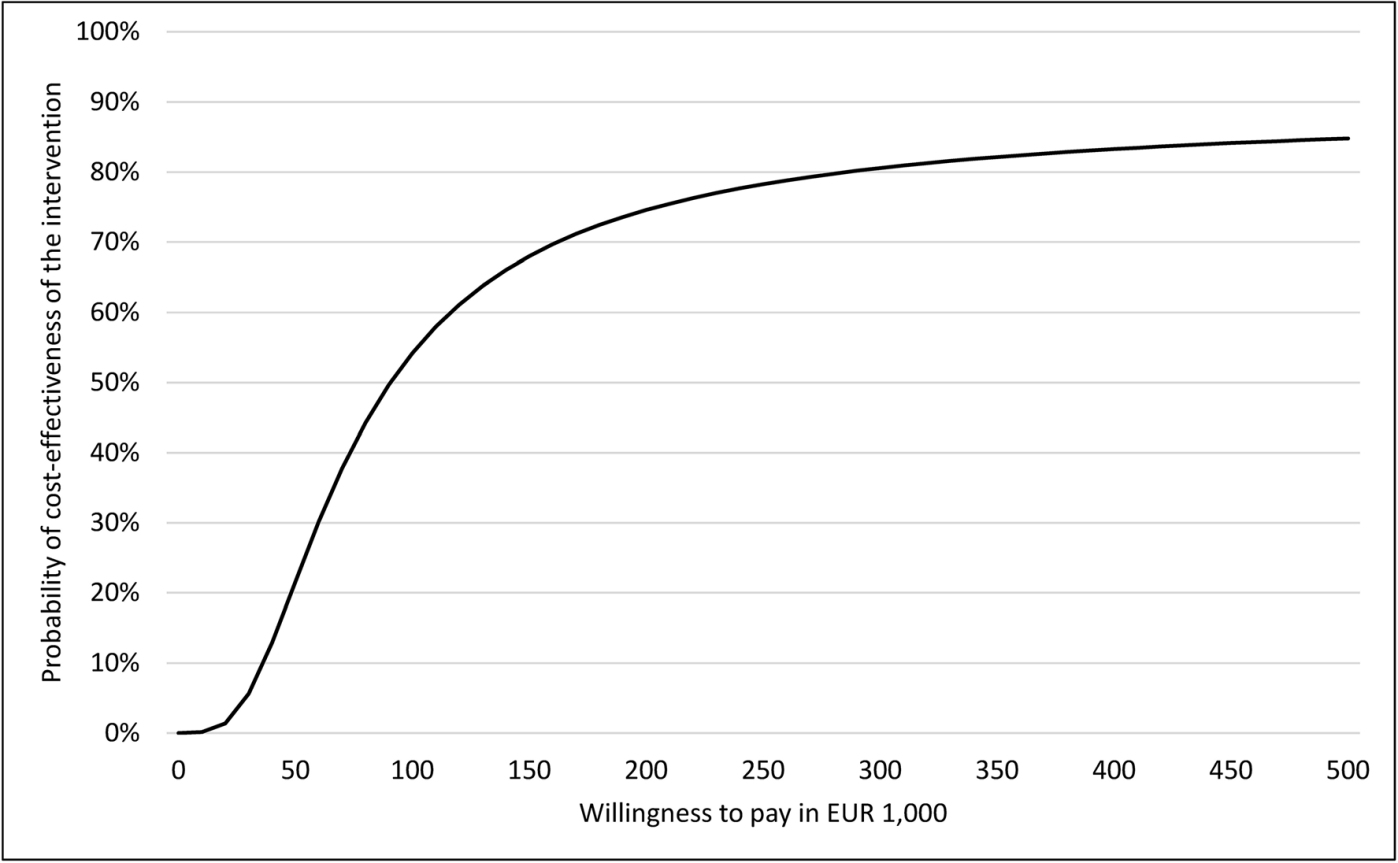
Cost-effectiveness acceptability curves of costs per fracture-free year of survival

The CEAC is displayed in Figure 1. At a WTP of 0€, the probability of cost-effectiveness was 0%. The probability equals 50% at a WTP of 90,000€, and seems to be steady at 85% at a WTP of 480,000€.

## DISCUSSION

OFRA was shown to prevent fragility fractures. Health-care costs decreased, except for DXA bone density measurement and osteoporotic medication. Total costs including implementation costs increased. Cost-effectiveness could not be shown within 1-year follow-up.

A differentiation between costs of preparation and operation is reasonable, as only the latter will occur to maintain the program. An additional analysis with only operational costs did not change the results considerably. Driving cost categories are staff costs for coordination, telecenters and prevention managers, all main elements of OFRA.

The health outcomes were investigated in detail in another publication.^13^ Fractures other than femoral, mortality, and nursing home admission did not significantly differ between groups. Both exercises^6,7,24^ and treatment of osteoporosis may have beneficial effects on fractures according to literature.^8^ However, approaches combining both measures are rare^25^ and cost-effectiveness has not yet been evaluated.

OFRA was based on a case-finding approach; thus, a large share of initially addressed individuals did not participate in one of the components of OFRA. This intention-to-treat approach induced a dilution effect which decreases the overall efficacy. By addressing particularly vulnerable risk groups, the uptake rate of OFRA components could be improved. This may increase efficacy.

The 1-year follow-up period may be too short to investigate long-term effects. Probably, a longer follow-up would increase the effectiveness. Yet, a decrease of femoral fractures was observed. When focusing only on these fractures, the probability of cost-effectiveness may increase.

OFRA may not only aim at reducing fragility fractures but additionally motivate regular exercise, a healthy lifestyle, and a general education on prevention to maintain physical abilities. OFRA may have increased the number of social interactions and decreased the number of falls not resulting in fractures. However, these factors were not considered in our analysis.

This study has some limitations. The choice of intervention districts may not be representative of the whole of Germany, as they were limited to rural districts. An implementation of OFRA in the eastern part of Germany was not possible due to a different infrastructure. The individuals were insurants of the SVLFG, who are people working in agriculture, gardening, and forestry. Therefore, the representativeness of the study may be limited to a rural population working in agriculture, gardening, and forestry. Furthermore, the payer perspective on costs does not cover all costs actually occurring. Particularly, costs of informal care and productivity losses due to sick leave were not covered. However, we assume a large proportion of persons being retired due to older age.

To name some strengths, our study was based on a large dataset of health insurance claims data. The intervention targeted a large German population and was implemented close to real-world conditions, as opposed to highly selected randomized controlled trials comprising a small population observed in non-daily conditions. The evaluation, therefore, has high external validity, and OFRA could be readily continued and further implemented.

## CONCLUSION

OFRA aimed at preventing fragility fractures in older adults living in rural areas in Germany by offering exercise classes, examination of bone health, and consultation on safety adjustments in the living environment. OFRA increased the fracture-free time of survival and improved examination and treatment of bone health. However, the costs for OFRA exceeded savings due to reduced health-care utilization. We found an unfavorable ratio of incremental costs and effectiveness. However, OFRA likely had further, unobserved effects on a physical and psychosocial level.
